# Serial magnetic resonance imaging findings during severe attacks of familial hemiplegic migraine type 2: a case report

**DOI:** 10.1186/s12883-021-02201-z

**Published:** 2021-04-21

**Authors:** David Fear, Misha Patel, Ramin Zand

**Affiliations:** 1 Geisinger Commonwealth School of Medicine, PA Scranton, USA; 2 Department of Neurology, Neuroscience Institute, Geisinger Health System, 100 N Academy Ave, PA Danville, USA

**Keywords:** ATP1A2, FHM, MRI, Hemiplegic, Migraine

## Abstract

**Background:**

Hemiplegic migraines represent a heterogeneous disorder with various presentations. Hemiplegic migraines are classified as sporadic or familial based on the presence of family history, but both subtypes have an underlying genetic etiology. Mutations in the *ATP1A2* gene are responsible for Familial Hemiplegic type 2 (FHM2) or the sporadic hemiplegic migraine (SHM) counterpart if there is no family history of the disorder. Manifestations include migraine with aura and hemiparesis along with a variety of other symptoms likely dependent upon the specific mutation(s) present.

**Case presentation:**

We report the case of an adult man who presented with headache, aphasia, and right-sided weakness. Workup for stroke and various infectious agents was unremarkable during the patient’s extended hospital stay. We emphasize the changes in the Magnetic Resonance Imaging (MRI) over time and the delay from onset of symptoms to MRI changes in Isotropic Diffusion Map (commonly referred to as Diffusion Weighted Imaging (DWI)) as well as Apparent Diffusion Coefficient (ADC).

**Conclusions:**

We provide a brief review of imaging findings correlated with signs/symptoms and specific mutations in the *ATP1A2* gene reported in the literature. Description of the various mutations and consequential presentations may assist neurologists in identifying cases of Hemiplegic Migraine, which may include transient changes in ADC and DWI imaging throughout the course of an attack.

## Background

Migraines affect more than 10 % of the population worldwide [1]. Migraines are clinically categorized into subtypes, dependent on the presence of and types of associated auras. Hemiplegic migraine (HM) is a subtype of migraine with aura, in which the aura has motor involvement, normally hemiparesis [2]. HM can be further classified as familial (FHM) if at least one first-degree or second-degree relative also meets the criteria for HM; otherwise it is classified as sporadic [3]. Currently, three genes have been identified to be associated with FHM: *CACNA1A* (FHM1), *ATP1A2* (FHM2), and *SCN1A* (FHM3) [2]. Penetrance for FHM 1, 2, and 3 appears to be high and is estimated to be approximately 80 % [2, 4, 5]. Data on the prevalence of FHM is lacking, but one study of a Danish population found it to be 0.01 % [6].

Patients with FHM typically present with their first attack within the first two decades of life. During acute attacks, many patients are initially worked up for possible stroke due to the associated hemiparesis [6, 7]. Within FHM2, there is immense variability in the clinical presentation, duration of attacks, and imaging findings. Some authors have reported perfusion computed tomography (CT) images that show hypoperfusion, others reported hyperperfusion, and some reported both throughout the attack [8, 9]. Magnetic Resonance Imaging (MRI) findings seem to vary depending on the FHM type. Cortical hyperintensities and swelling on MRI have been well described for FHM1 patients. Less is known about MRI findings for FHM2 [10].

Here, we describe a patient who presented to the emergency room with a headache, hemiparesis, and aphasia. During his extended hospital stay, we report a series of MRI studies obtained over the course of the acute attack. Genetic testing revealed a missense mutation in the *ATP1A2* characteristic of FHM2.

## Case presentation

 A 25-year-old right-handed man presented to the emergency department with a headache, right-sided weakness, confusion, and aphasia which began the previous night (day 0). On day 0, the patient awoke late in the afternoon with a headache and some confusion according to his family. The patient then went back to sleep until the following morning. Upon awakening, the patient exhibited right-sided weakness and difficulty speaking in addition to the headache and confusion. His mother also reported the patient was nauseous and had episodes of vomiting throughout the night. The patient had a questionable history of migraine with prolonged aura. Prior to the onset of the headache, the patient was in his usual state of health. Unique to this experience, which prompted his parents to bring him to the emergency room, was the *persistence* of headaches, hemiplegia, and difficulty speaking. A level II stroke alert prompted a CT of his head and Computed Tomography Angiography (CTA) of his head and neck; both were negative for any signs of a stroke, stenosis, or occlusion. The consulting neurologist diagnosed the patient with atypical migraine since the symptoms were similar to those previously experienced by the patient in prior attacks. The patient was instructed to return for possible MRI of the brain if symptoms persisted and was referred to an outpatient neurology visit for follow-up. The patient was discharged with a diagnosis of atypical migraine and with instructions for follow-up or to return to the emergency department if symptoms worsened. The patient returned the following day (day 2) due to the persistence of symptoms. Thus, the patient was admitted for observation with right-sided weakness, aphasia, and fever. At 5:00 PM on day two, the patient recorded a temperature of 100.9℉. During the next several days, the patient’s temperature ranged from 98.3℉ − 102.6℉. The patient was febrile to a temperature max of 102.6℉ recorded on day 4 at 4:00 AM. The patient’s fever subsequently broke by at 6:00 AM of day 4. Of interest, the patient reported two female cousins with similar symptoms.

**Fig. 1:**
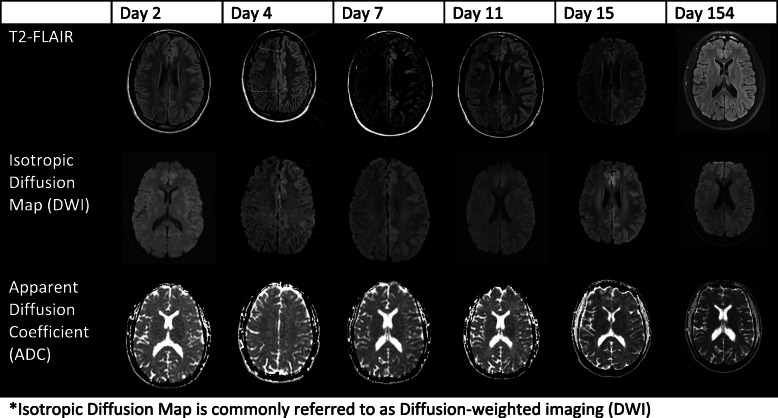
MRI obtained on day 2 did not reveal any acute intracranial abnormality. However, scattered, non-enhancing punctate T2-FLAIR hyperintensities in subcortical white matter were present bilaterally in the frontal lobes. Day 4: Diffuse DWI and T2-FLAIR hyperintensities throughout the left cerebral hemisphere cortices, most pronounced over the lateral convexity. Possible associated T2 shinethrough rather than restricted diffusion. Associated sulcal effacement from gyral swelling. Day 7:Diffuse DWI and T2-FLAIR hyperintensities throughout the left cerebral hemisphere cortices similar to the MRI findings obtained on day 4. Subtle hypointensity on ADC in left cerebral hemisphere cortices and posterior temporal lobe. Day 11: Diffuse DWI and T2-FLAIR hyperintensities throughout the left cerebral hemisphere cortices similar to previous MRI findings. Mild hypointensity on ADC in the left cerebral hemisphere corticesand temporal lobe is more prominent compared to previous MRIs. Day 15: Diffuse DWI and T2-FLAIR hyperintensities throughout the left cerebral hemisphere cortices similar to previous MRIs. Mild hypointensity on ADC in left cerebral hemisphere cortices is even more prominent compared to previous MRIs. Day 154:Previously demonstrated left cerebral hemisphere cortical swelling and restricted diffusion has resolved. No new parenchymal signal abnormality or abnormal enhancement. Scattered non-enhancing punctate T2-FLAIR hyperintensities were present in subcortical white matter of bilateral frontal lobes similar to the first MRI

 The neurological findings and fever prompted empiric treatment for meningitis. A lumbar puncture (LP) was ordered to assess for infectious etiologies. The patient was unable to be interviewed due to aphasia. History obtained from the patient’s parents revealed the patient had been working outside at a car shop and taking several stimulant weight loss supplements in the days immediately prior to admission. The clinical exam showed an alert, dysarthric patient with global aphasia and weakness in the right upper and lower extremities. The first MRI was obtained approximately two days after symptom onset. T2-fluid-attenuated inversion recovery (FLAIR) showed no signs of acute intracranial abnormality but did show scattered hyperintensities in the white matter of frontal lobes bilaterally (Fig. 1).

On the day following his admission (day 3), the results of the patient’s LP showed clear, colorless fluid which was negative for xanthochromia. Glucose and protein were within normal limits while white blood cells and red blood cells showed 2 cells/mm^3^ and 1 cells/mm^3^ respectively. Polymerase Chain Reaction (PCR) was negative for enterovirus, herpes-simplex virus (HSV) 1 and 2, and cryptococcal antigens. The culture of the cerebrospinal fluid (CSF) was ultimately negative for bacterial species, but his fever persisted. He underwent seizure-like activity which prompted treatment with 1 mg Intravenous (IV) Lorazepam and subsequent resolution of the episode. A loading dose of 1000 mg of IV Levetiracetam followed by 500 mg twice a day as well as further workup was ordered. Another MRI was obtained the day following his seizure-like activity (day 4), which showed diffuse diffusion-weighted imaging (DWI) and T2/FLAIR hyperintensities throughout the left cerebral hemisphere cortices. Electroencephalogram (EEG) was ordered and returned abnormal results indicative of global central nervous system (CNS) dysfunction and focal dysfunction of the left hemisphere. Encephalitis was suspected, but the exact etiology was still unknown.

Empiric treatment for bacterial meningitis was discontinued on day 5 following negative LP and advice from infectious disease consultations. With a tick-borne molecular panel still pending, empiric treatment with doxycycline was advised. HIV, fungal, bacterial, and viral agents all returned negative. On day 7, the patient experiences a generalized tonic-clonic (GTC) seizure which resulted in the decision to start long-term EEG and repeat MRI. The repeat MRI showed similar findings to the previous MRI - T2/FLAIR hyperintensity of the left cerebral cortex without enhancement and associated sulcal effacement. At this time, the differential was still broad including encephalitis of viral, neoplastic, autoimmune, or idiopathic origins. A steroid regimen was employed.

Figure 1 highlights the MRI findings throughout the course of the patient’s hospital stay as well as the resolution and follow-up visits. Day 11 was notable for more prominent hypointensity on Apparent Diffusion Coefficient (ADC) in the left cerebral hemisphere cortices and temporal lobe. MRI on day 15 revealed even more prominent hypointensity on ADC. Continuous EEG recording was started on day 12 and continued for 9 days. The findings from EEG were largely non-specific but included left-hemispheric slowing with mild diffuse slowing. By the end of the EEG recordings, the left hemispheric attenuation had largely resolved.

To rule out germ cell tumor, CT of the chest, abdomen and pelvis was obtained on day 16 and showed no evidence of neoplasm. Despite the lack of success identifying a causal agent, the patient showed signs of clinical improvement with the persistence of the aphasia. Due to the negative LPs, the negative pan CT, and non-specific EEG findings, the differential now included limbic encephalitis, autoimmune, prion disease, Transient Headache and Neurologic Deficits With Cerebrospinal Fluid Lymphocytosis (HaNDL syndrome) and last, but not least, the Rasmussen syndrome. LP, anti-Hu/Ma, anti-Ta, anti-NR1, anti-AQP4, anti-CASPR2, anti-LGI1, anti-Yo, oligoclonal bands, ANA, anti-double stranded DNA, complement C3 and C4, SSA-SSB antibody, RNP-SM antibody, encephalitis antibody evaluation (ANNA-1/anti-Hu, ANNA-2/anti-Ri, ANNA-3, PCA-1/anti-Yo, PCA-2, PCA-TR/anti-Tr, AGNA/anti-SOX1, Amphiphysin Ab, anti-CRMP5/anti-CV2, anti-GAD65, anti-MA2/Ta anti-myelin, AQP4/Ab/NMO-IgG), an epilepsy autoimmune evaluation (AChR Ganglionic Neuronal Ab, AMPA-R Ab, Amphiphysin Ab, AGNA-1, ANNA-1/anti-Hu, ANNA-2/anti-Ri, ANNA-3, CASPR2-IgG, CRMP-5-IgG, DPPX Ab, GABA-B-R Ab, anti-GAD65, anti-GFAP, LGI1-IgG CBA, mGluR1 Ab, NMDA-R Ab, N-Type Calcium Channel Ab, P/Q-Type Calcium Channel Ab, PCA-2, PCA-Tr), a neurology antibody order (ANNA-1/anti-Hu, ANNA-2/anti-Ri, PCA-1/anti-Yo, PCA-TR/anti-Tr, AGNA/anti-SOX1, anti-Ma2/Ta, anti-Zic4, anti-CRMP5/anti-CV2, amphiphysin Ab, anti-GAD65) and protein 14-3-3 were ordered as part of the follow-up. The decision was made to start the patient on a course of IVIG while additional investigations were still pending. The patient showed improvement after starting the IVIG and was discharged on multiple medications including lamotrigine, levetiracetam, magnesium chloride, riboflavin, valproate sodium and instructions with close follow-ups. At the outpatient follow-up with neurology, the patient showed significant improvements in language, showed no stroke-like symptoms, and denied any seizure-like activity. The rest of the laboratory investigations were unremarkable except genetic testing. The analysis of four genes (*ATP1A2, CACNA1A, SCN1A, PRRT2*), was positive for a heterozygous, AD mutation in *ATP1A2* (c.2936 C > T p. P979L). The patient had a recurrence of seizure which resulted in another short hospital stay.

## Discussion and conclusions

The *ATP1A2* gene mutation found in FHM2 patients affects an ATPase found primarily in the CNS, specifically in hippocampal astrocytes and pyramidal cells. Dysfunction of this protein is thought to lead to increased extracellular K^+^, intracellular Na^+^, and intracellular Ca^2+^, all contributing to cortical spread depression which has been hypothesized to be the pathophysiological mechanism of the migraine aura [9, 11–14]. Various mutations in the *ATP1A2* gene lead to phenotypically heterogeneous presentations of FMH2 which have been previously described [9]. The various presentations of mutations in *ATP1A2* often require ruling out life-threatening causes of hemiparesis, such as stroke and infectious meningoencephalitis [7, 15]. One common finding amongst FHM2 patients is the presence of a fever [6, 9, 10, 15], also frequently found in FHM1 patients [14, 16].

Table 1 includes clinical presentations, imaging findings, and genetic testing if performed for patients with hemiplegic migraines with an *ATP1A2* mutation undergoing severe attacks published in the last ten years [6, 9, 10, 15, 17–24]. Perfusion studies on FHM2 patients have demonstrated both hypo- and hyperperfusion. This could potentially be explained by a biphasic change in cerebral blood flow experienced during acute attacks [8]. A previous theory suggested that the aura phase was related to hypoperfusion and headache with hyperperfusion [7]. However, the finding that aura symptoms can persist when imaging studies demonstrate hyperperfusion opposes this theory. More research is needed to understand the relationship, if any, between perfusion changes and clinical signs/symptoms of FHM patients. Dynamic MRI changes during attacks are demonstrated in both previous reports as well as this patient.

**Table 1:** Clinical symptoms and imaging findings in patients with ATP1A2 mutations

Paper	Patient #^a^	Mutation	Attack #	Days after symptom onset^b^	Symptoms at time of imaging (symptoms in between imaging)	Imaging Type^e^	Imaging Findings
Asghar et al. (2012) [17]	2	Leu to Pro, upstream 1025 bp on the ATP1A2	#1	3	Lethargy, altered mental status, gait difficulties, ataxia, monoparesis, expressive aphasia	MRI	Diffuse cortical edema in affected hemisphere
				~ 3 weeks	Unknown	MRI	Unremarkable
Blicher et al. (2016) [6]	1		#1	0	Headache, nausea, photophobia, aphasia	T2-FLAIR, DWI; Perfusion MRI	Nonspecific white matter lesions; hypoperfusion
				12	Normal motor function, persistent aphasia/aura (focal seizures)	T2-FLAIR, DWI; APT/CEST-MRI	Hyperintense cortical gray matter; pH decrease in white matter
Guedj et al. (2010) [18]	1	p.935-940del ins Ile	#1	1	^c^Hemiplegia, hypoesthesia, dysarthria, aphasia, visual and sensory disturbances, headache, photophobia, phonophobia, nausea	MRI	Unremarkable
				78	Monoparesthesis	MRI	Unremarkable
Hermann et al. (2013) [19]	2	p.Pro979Leu	#1	Unknown	^c^Scotoma, numbness, hemiparesis, headache, fever, seizures	cMRI	Unremarkable
						DWI; MRA	Diffuse cortical swelling, abnormal cortical diffusion; “string and beads” arteries
Iizuka et al. (2012) [15]	1		#1	2	^c^Confusion, hemiparesis, visual hallucination, psychiatric symptoms	SPECT	Decreased
				3		DWI	Unremarkable
						MRA	Prominently increased in Middle Cerebral Artery (MCA)
			#2	2	^c^Confusion, hemiparesis, aphasia, visual-field defect, visual hallucination, psychiatric symptoms	DWI	Unremarkable
						SPECT	Increased
				3		MRA	Prominently increased in MCA
				4		DWI	Unremarkable
						T2-FLAIR; SPECT	Mild cortical edema, CSF enhancement; increased
			#3	3	^c^Confusion, hemiparesis, aphasia, visual-field defect, psychiatric symptoms	T2-FLAIR, DWI	Unremarkable
						SPECT; MRA	Increased; mildly increased in MCA
			#4	1	^c^Confusion, hemiparesis, aphasia, visual-field defect, visual hallucinations, psychiatric symptoms	T2-FLAIR, DWI	Unremarkable
						MRA	Mildly decreased in MCA
				2		SPECT	Increased
			#5	2	^c^Confusion, hemiparesis, visual-field defect, visual hallucinations, psychiatric symptoms	T2-FLAIR, DWI	Unremarkable
						SPECT	Decreased
			#6	1	^c^Delirium, hemiparesis, aphasia, visual-field defect, visual hallucinations, auditory hallucinations, psychiatric symptoms	T2-FLAIR, DWI	Unremarkable
						SPECT	Decreased
	2		#1	2	^c^Confusion, hemiparesis, visual-field defect, visual hallucination, psychiatric symptoms	T2-FLAIR, DWI	Unremarkable
						SPECT, MRA	Increased in MCA
			#2	2	^c^Confusion, hemiparesis, visual-field defect, visual hallucination, psychiatric symptoms	T2-FLAIR, DWI	Unremarkable
						MRA	Mildly increased in MCA
				3		SPECT	Increased
Martinez et al. (2016) [9]	1	p.Thr364Met	#1	0	Hemiparesis, aphasia, headache, nausea, photophobia, gaze preference	perfusion CT	Hypoperfusion
				3	Worsened aphasia, somnolence, fever	DWI	Unremarkable
Murphy et al. (2018) [20]	2	c.Ala2324Gly in exon 17 p.Tyr775Cys	#1	5^d^	Headache, photophobia, movement sensitivity, nausea, vomiting, hemiparesis, somnolence, positive Babinski	T2-FLAIR	Cortical edema in affected hemisphere
				12^d^		T2-FLAIR, DWI, T1	Cortical edema in affected hemisphere with associated sulcal effacement, mass effect, restricted diffusion, leptomeningeal enhancement over affected mesial temporal lobe
Rispoli et al. (2019) [21]	1	p.Gly954Arg	1	3	^c^Migraine, vomiting, hemiparesis, paraesthesias, ataxia, diplopia, acute confusion	T2-FLAIR; MRA	Hyperintensities with mild cortical swelling, sulcal effacement, restricted diffusion, contrast enhancement; lower signal
				10		T2-FLAIR	Persistent cortical swelling
Roth et al. (2018) [10]	1	p.Arg908Gln	#1	11	Hemiplegia, somnolence	T2-FLAIR, DWI	Swelling and cortical hyperintensity
			#2	2	^c^Hemiplegia, aphasia, drowsiness	T2-FLAIR, DWI, cMRI	Unremarkable
				3		Perfusion CT	Increased
				9		T2-FLAIR, DWI	Swelling and cortical hyperintensity
				15		T2-FLAIR, DWI	Clear improvement
	2	p.Arg908Gln	#1	7	Symptom free (presented with headache, vomiting, photophobia, phonophobia, hemiparesis, aphasia)	T2-FLAIR; DWI	Mild cortical hyperintensity; cortical hyperintensity
	3	p.Ser220Leu	#1	5	Drowsiness (hemiparesis had resolved)	T2-FLAIR; DWI	Mild swelling and cortical hyperintensity; mild cortical hyperintensity
			#2	6	Symptoms almost resolved (hemihypesthesia)	Perfusion CT	Increased
	4	p.Arg908Gln	#1	2	Mild aphasia (hemiparesis had resolved)	T2-FLAIR, DWI	Unremarkable
Schwarz et al. (2018) [22]	1	p.Thr364Met	#1	1	Hemiparesis, speech disturbances, headache, fever, confusion, anesthesia	CT, DWI	Unremarkable
						MRI; FLAIR	Prominent draining sulcal veins; minimal diffuse thickening in affected cortex
				9	Unspecified clinical improvement	DWI	Unremarkable
						MRI; FLAIR	Hyperperfusion, reduced draining sulcal veins; minimal diffuse thickening
Toldo et al. (2010)	1	c.1091 C > T (p.Thr364Met) in heterozigosis on exon 9	#1	0	Consciousness impairment, fever, motor deficit, aphasia	MRI	Unremarkable
				4		MRI- FLAIR; DWI	Cortical swelling in affected hemisphere; hyperintensity
				11	Motor deficit, aphasia	MRI-FLAIR; DWI	Progressive cortical swelling in affected hemisphere; hyperintensity
				15		Proton MRI Spectroscopy	Decreased N-acetylaspartate/creatine ratio in affected hemisphere
				27		99mTc-ECD SPECT	Marked hypoperfusion in affected hemisphere
				6 months	Resolved	MRI, SPECT	Unremarkable
Wilbur et al. (2017) [24]	1	p.Arg1008Trp	#1	Unknown, 1st image	Seizures without hemiparesis	MRI	Unremarkable
				Unknown, 2nd image	Fever, seizures, hemiparesis, unresponsiveness, eye deviation	MRI	Unremarkable
				Unknown, 3rd image	Lethargy, vomiting, fever, hemiparesis, seizures	MRI	Subtle atrophy, swelling, diffuse hyperintensities in affected hemisphere
Our patient	1	Pro979Leu	#1	1	Headache, hemiparesis, confusion, aphasia	CT	Unremarkable
				2	Headache, hemiparesis, confusion, aphasia, fever	DWI	
						T2-FLAIR	Nonspecific nonenhancing bilateral hyperintensities
				4, 7	Persistent symptoms (seizure)	DWI; T2-FLAIR	Diffuse low-level restricted diffusion; prolongation
				11		DWI, T2-FLAIR	Hyperintensities
				15		DWI	Hyperintensities
						T2-FLAIR	Diffuse cortical swelling, mild asymmetric hyperintensities

^a^Patient number corresponds to the number assigned to patient in original paper for easy reference

^b^Symptom onset based on our definition

^c^Summary of clinical symptoms during attack. Original paper did not detail which symptoms were present at time of imaging

^d^Days 1 and day 8 in hospital. The authors did not specify which symptoms resolved, if any, by the time of either imaging study

^e^*APT/CEST-MRI* amide proton transfer chemical exchange saturation transfer magnetic resonance imaging, *cMRI* cardiovascular magnetic imaging resonance, *DWI* diffusion-weighted, *MRI* FLAIR, fluid-attenuated inversion recovery, *MRA* magnetic resonance angiography, *MRI* magnetic resonance imaging, *SPECT* single-photon emission computed tomography

The symptoms experienced by our patient during the acute attack were not associated with MRI changes within the initial days of migraine onset. This is consistent with previous findings described by Roth et al. [10] which suggested that changes in imaging tend to be found later during the course of an attack. This dissociation of clinical symptoms and imaging findings is not unique to FHM patients and is also notably found in Reversible Cerebral Vasoconstriction Syndrome [25]. In our patient, cortical swelling and restricted diffusion in the left cerebral hemisphere was appreciated by day 4 of the attack and persisted throughout the series of MRI images obtained during the course of the patient’s hospital stay. A unique feature of our patient’s imaging findings is the hypointensities observed on ADC. This finding was not reported in the studies included in Table 1. Belvís at al. [26] describe transient changes in ADC seen during a persistent visual aura which seems similar to the findings observed in our patient. Future studies of the condition may benefit from the inclusion of transcranial doppler or angiography to further observe these reversible vasospasms.

Consistent with similar cases described in literature, these imaging changes resolved overtime. The reversible encephalopathy that can occur during these migraine attacks has been described by the studies summarized in Table 1, which illustrates the diversity of findings observed during attacks in patients with various mutations in *ATP1A2*. The patient presented here provides a chance to examine five MRIs obtained throughout the course of the prolonged attack as well as an MRI obtained after resolution of symptoms. The importance of this paper is to describe not only the phenotypic changes, but also the imaging findings across the course of attacks in patients with various mutations in the *ATP1A2* gene (FMH2). When possible, we present the mutation, clinical features and imaging findings found during various days into each described encounter. This attempt to combine and present findings may be a useful resource for clinicians to appreciate the heterogeneous nature of the disease described from the genetic, clinical and imaging perspectives in various mutations of *ATP1A2*.

Many patients experiencing a FHM attack are treated with various antivirals, antibacterials, antiepileptics, corticosteroids, and migraine medications. Unique to our patient’s treatment plan was the use of IVIG for presumed autoimmune encephalitis as encephalitis can present similarly. IVIG was started on day 18 and symptoms improved on day 19. Granted, this observation cannot be solely attributed to IVIG targeting an immune etiology as it may represent a naturally resolving attack, especially given the original baseline health, timeline of symptoms, and the genetic confirmation of FHM. Immunological interventions have historically not been the treatment of choice in FHM patients. Here they were used after bacterial meningitis had been ruled out, but while various investigations were still pending.

Multiple mutations and resultant phenotypic findings have been described in patients with FHM2. Here, we summarize a number of studies that describe imaging findings found at the time of various clinical signs and symptoms with respect to the specific mutation present in *ATP1A2.* We also present transient changes in MRI sequences during an acute attack in this FHM2 patient, which highlights some nuanced findings such as hypointensities present on ADC imaging.

## Data Availability

The dataset referenced for this study can be de-identified and made available by request to the corresponding author with appropriate data usage agreements.

## Abbreviations

HM: Hemiplegic Migraine
FHM: Familial Hemiplegic Migraine
SHM: Sporadic Hemiplegic Migraine
CT: Computed tomography
MRI: Magnetic resonance imaging
CTA: Computed tomography angiography
LP: Lumbar puncture
FLAIR: Fluid-attenuated inversion recovery
PCR: Polymerase chain reaction
HSV: Herpes-simplex virus
CSF: Cerebrospinal fluid
IV: Intravenous
DWI: Diffusion-weighted imaging
EEG: Electroencephalogram
CNS: Central nervous system
GTC: Generalized tonic-clonic
ADC: Apparent diffusion coefficient
HaNDL: Transient headache and neurologic deficits with cerebrospinal fluid lymphocytosis
ANNA: Antineuronalnuclear antibody type 1
anti-PCA: Purkinje cell cytoplasmic antibody
AGNA: Anti-glianuclear antibody
CRMP: Collapsinresponse mediator protein
GAD: Glutamicacid decarboxylase
AQP4: Aquaporin-4
AMPA: Alpha-amino-3-hydroxyl-5-methyl-4-isoxazolepropionicacid
CASPR: Contactin-associatedprotein
DPPX: Dipeptidyl-peptidase-likeprotein 6
GABA-B-R: Gamma-aminobutyricacid-B receptor
GFAP: Glialfibrillary acidic protein
LGI-1: Leucine-richglioma-inactivated
mGluR1: Metabotropic glutamate receptor 1
NMDA-R: N-methyl-d-aspartatereceptor
APT/CEST-MRI: Amideproton transfer chemical exchange saturation transfer magnetic resonanceimaging
cMRI: Cardiovascularmagnetic imaging resonance
MRA: Magnetic resonance angiography
SPECT: Single-photon emission computed tomography
